# Bone Regeneration and Remodeling within a Unidirectional Porous Hydroxyapatite Bone Substitute at a Cortical Bone Defect Site: Histological Analysis at One and Two Years after Implantation

**DOI:** 10.3390/ma8084884

**Published:** 2015-07-30

**Authors:** Masashi Iwasashi, Toru Funayama, Arata Watanabe, Hiroshi Noguchi, Toshinori Tsukanishi, Yasushi Suetsugu, Takeshi Makihara, Naoyuki Ochiai, Masashi Yamazaki, Masataka Sakane

**Affiliations:** 1 Department of Orthopaedic Surgery, Tsukuba Medical Center Hospital, 1-3-1 Amakubo, Tsukuba, Ibaraki 305-8550, Japan; E-Mail: iwasashi-m@hotmail.co.jp; 2 Department of Orthopaedic Surgery, Kenpoku Medical Center, Takahagi Kyodo Hospital, 1006-9 Agehocho, Kamitetsuna, Takahagi, Ibaraki 318-0004, Japan; E-Mails: funatoru3@gmail.com (T.F.); tsukanishicareer@yahoo.co.jp (T.T.); 3 Department of Orthopaedic Surgery, Ichihara Hospital, 3681 Ohzone, Tsukuba, Ibaraki 300-3295, Japan; E-Mail: arata-new@knd.biglobe.ne.jp; 4 Tsukuba Central Hospital, 1589-3 Kashiwada-Cho, Ushiku, Ibaraki 300-1211, Japan; E-Mail: noguhiro0164@tsukuba-seikei.jp; 5 Biomaterials Unit, National Institute for Materials Science, 1-1 Namiki, Tsukuba, Ibaraki 305-0044, Japan; E-Mail: suetsugu.yasushi@nims.go.jp; 6 Department of Orthopaedic Surgery, University of Tsukuba, 1-1-1 Tennodai, Tsukuba, Ibaraki 305-8575, Japan; E-Mails: makihara@tsukuba-seikei.jp (T.M.); ochiainaoyuki@ob.md.tsukuba.ac.jp (N.O.); masashiy@md.tsukuba.ac.jp (M.Y.)

**Keywords:** unidirectional porous hydroxyapatite (UDPHAp), osteogenesis, bone remodeling, implantation, cortical defect

## Abstract

Unidirectional porous hydroxyapatite (UDPHAp) is an artificial bone substitute with a unique microstructure consisting of 100–300-µm oval pores that present the material unidirectionally. UDPHAp has a compression strength of 14 MPa and a porosity of 75%, which promotes cell migration and capillary formation within the material. Despite these advantageous properties, bone remodeling and bone formation with UDPHAp remain unclear. To examine long-term remodeling and differences in bone formation based on the defect site, trapezoidal prism-shaped UDPHAp blocks were implanted into rectangular-shaped cortical bone defects in the proximal tibia of Japanese white rabbits. Histological analysis performed at 52 and 104 weeks after implantation revealed that bone and capillaries had formed within the implanted UDPHAp material. Bone formed within the UDPHAp implanted in the cortical defect of rabbit tibia and remodel up to two years. The percentage of new bone area within UDPHAp was larger in cortical lesions than that in medullary lesions. These findings suggest that UDPHAp is a promising material for the repair of non-critical-sized cortical bone defects.

## 1. Introduction

The development of calcium phosphate ceramics and other related biomaterials for bone grafting has allowed for increased control of the resorption and bone substitution processes at graft sites. In particular, porous hydroxyapatite is widely used as a bone substitute due to its good osteoconductivity and bioinert properties. However, despite these advantages, porous hydroxyapatite may be displaced at defect sites and lack structural integrity after implantation due to poor bone formation within the material. Increasing the porosity and interconnectivity of the underlying porous structure and designing nanocomposite scaffolds have been shown to promote bone ingrowth into the implanted material [1,2,3,4].

**Figure 1:**
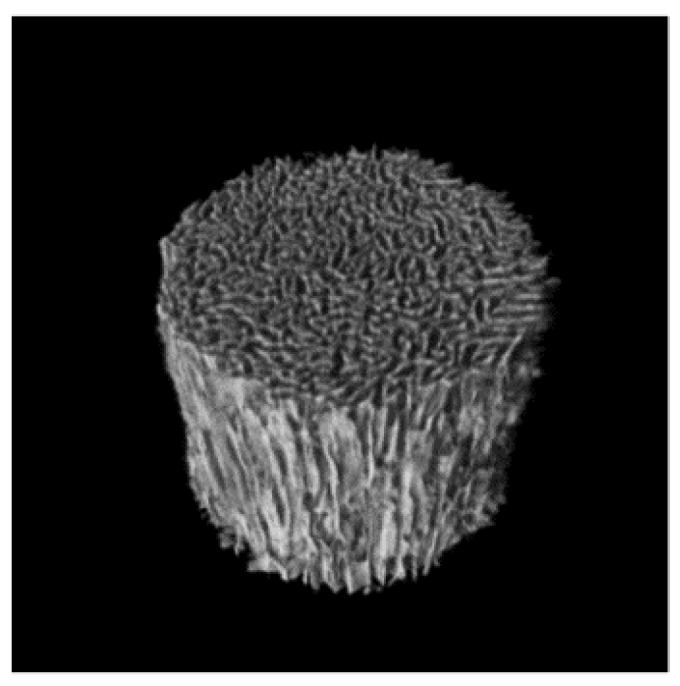
Pore architecture of UDPHAp. The pores are unidirectionally orientated parallel to the long axis of the cylinder.

Using this approach, we previously developed an artificial bone substitute material, termed unidirectional porous hydroxyapatite (UDPHAp), which contains oval pores (100–300 µm in diameter) that penetrate unidirectionally through the material (Figure 1) [5]. Due to this unique microstructure, the implantation of UDPHAp into a rabbit tibial defect model results in the rapid formation of capillaries and new bone for up to 12 weeks [6]. Although these properties suggest that UDPHAp is potentially suitable for the repair of large bone defects in the clinical setting, long-term remodeling and effects of surrounding environments on bone formation within the substitute were unclear.

The purpose of the present study was to further investigate long-term bone formation and remodeling within UDPHAp after implantation into non-critical size cortical bone defects within rabbit tibia after one and two years using the same cortical defect model in rabbits. Bone formed in the UDPHAp remodeled and adapted by mechanical and chemical stimulus from surrounding tissues.

## 2. Results and Discussion

**Figure 2:**
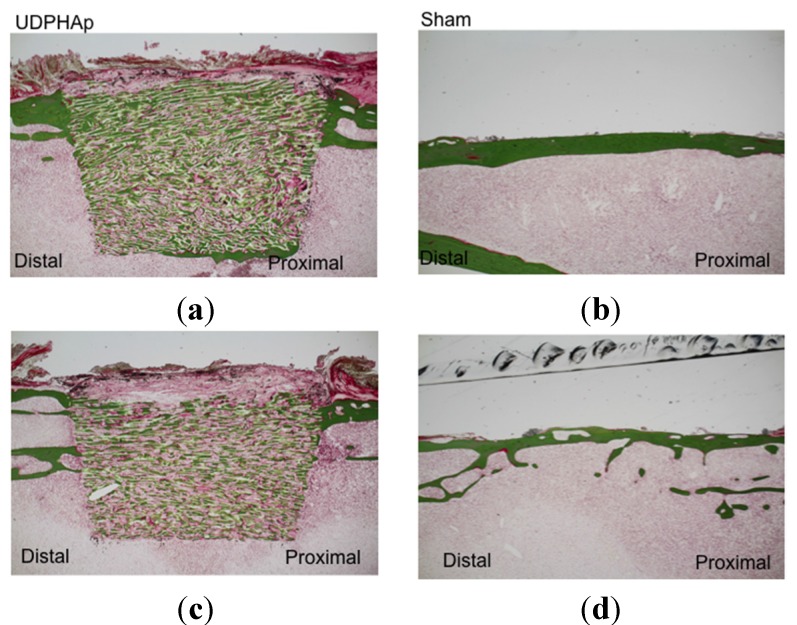
Histological findings of undecalcified sections stained with Villanueva-Goldner stain at 52 weeks after UDPHAp transplantation (**a** and **c**). Sham-operated specimens are also shown (**b** and **d**). Bone is stained in green. The cortical defect was 7 mm in length.

**Figure 3:**
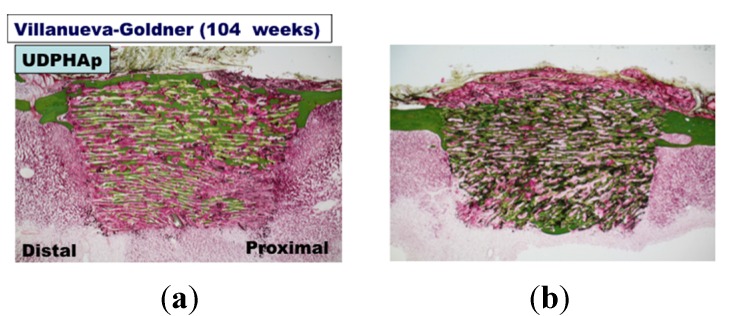

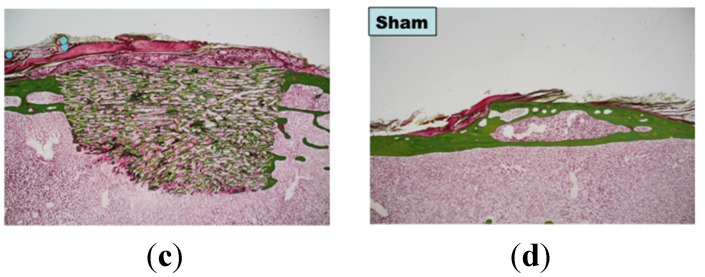
Histological findings undecalcified sections in Villanueva-Goldner staining at 104 weeks after UDPHAp transplantation (**a**, **b** and **c**). A sham-operated specimen is also shown (**d**). Bone is stained in green.

Undecalcified ground sections of UDPHAp were subjected to Villanueva–Goldner staining at 52 weeks (*n* = 2, Figure 2) and 104 weeks (*n* = 3, Figure 3) after implantation. The staining showed that new bone had formed along the UDPHAp pore walls and that the cortical bone and new bone were continuous with the surrounding bone. At both 52 and 104 weeks, bone formation was observed within the bone substitute. The edges of the substitute appeared sharp and uniform at 52 weeks, but were considerably less distinct by 104 weeks. In the sham-operated specimens, cortical bone defects were healed and filled with autologous bone (Figure 2 and Figure 3).

**Figure 4:**
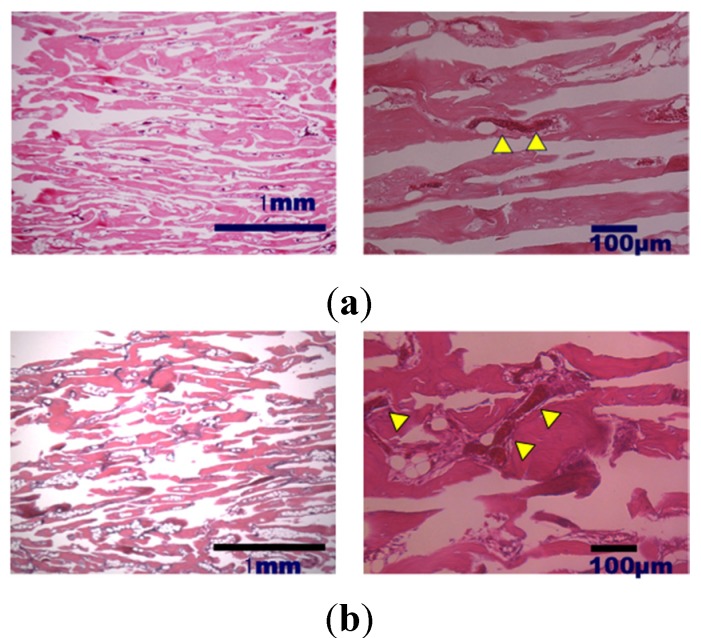
Histological analysis of decalcified specimens of UDPHAp by hematoxylin-eosin staining performed 52 weeks (**a**) and 104 weeks (**b**) after transplantation. Capillaries were observed at higher magnification (yellow arrow head).

**Figure 5:**
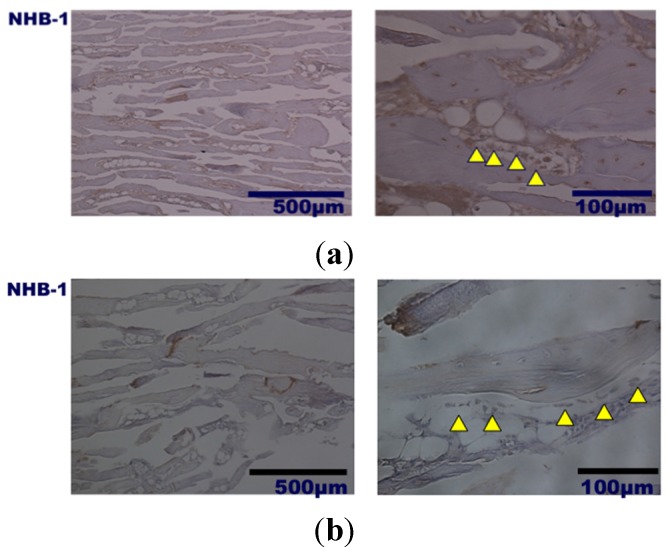
Histological findings of decalcified UDPHAp specimens by osteocalcin immunostaining at 52 weeks (**a**) and 104 weeks (**b**) after transplantation. Cells lined below formed bone (yellow arrow head) are positively stained.

**Figure 6:**
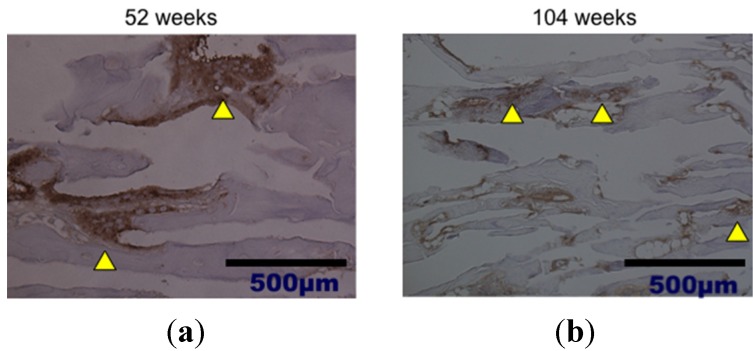
Cathepsin K immunostaining of osteoclast was performed at 52 weeks (**a**) and 104 weeks (**b**) after transplantation of UDPHAp. Cathepsin K-posive multinuclear cells (yellow arrow head) were predominantly located in medullae at 52 weeks.

**Figure 7:**
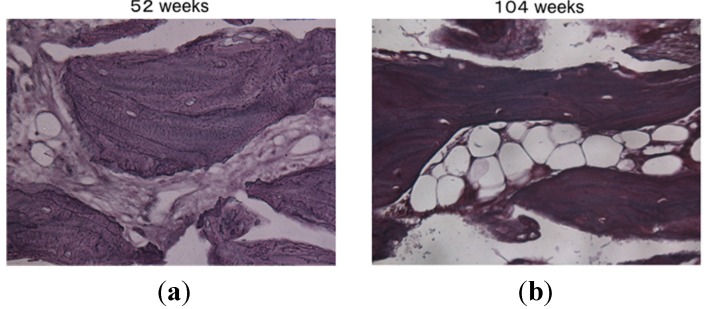
Bodian staining at 52 weeks (**a**) and 104 weeks (**b**) after transplantation of UDPHAp. The lamellar structure of the newly-formed bone was more developed at 104 weeks after UDPHAp transplantation.

Histological analysis of decalcified slices of the UDPHAp blocks stained with hematoxylin-eosin at 52 and 104 weeks after implantation revealed that capillaries had formed in the new bone tissue (Figure 4). In high-magnification images of HE-stained samples, fine hydroxyapatite crystals were clearly observed at 104 weeks. Immunostaining of osteocalcin revealed that clusters of osteoblasts were present within the UDPHAp bone substitute (Figure 5). Cathepsin K-positive multinuclear cells were also detected within the newly-formed bone, but were not observed in association with hydroxyapatite (Figure 6). Notably, more cathepsin K-positive areas were observed in the medullary luminal region of the defect site than the cortical bone region at both 52 and 104 weeks. In addition, fewer osteoclasts and osteoblasts were detected within the UDPHAp blocks at 104 weeks compared to the 52-week samples. Bodian staining at 52 and 104 weeks after UDPHAp transplantation detected the presence of both osteocytes and bone canaliculi (Figure 7). As can be seen in Figure 3 and Figure 4, the edges of the implanted specimens were relatively sharp at 52 weeks, whereas the regions of the bone substitute material at the interface with the medullary region of the host bone were partially resorbed.

**Figure 8:**
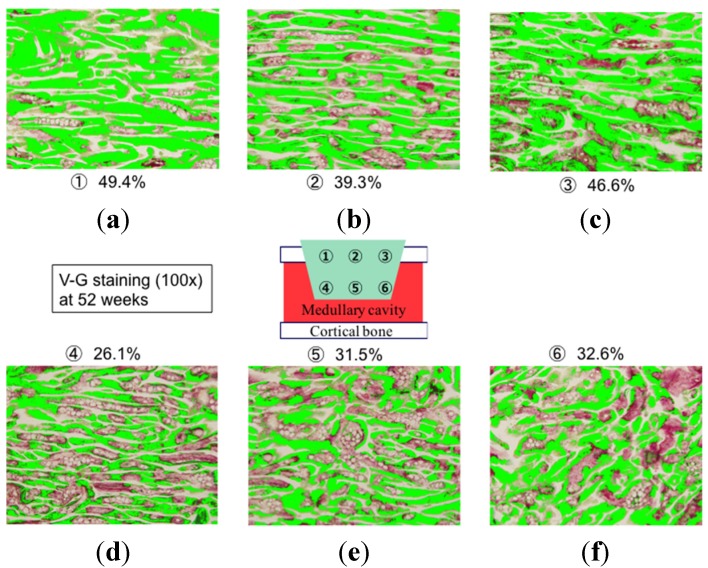
Histological findings undecalcified sections in Villanueva-Goldner staining at 52 weeks after UDPHAp transplantation. Bone is stained in green in the cortical (**a**, **b**, and **c**) and medullary (**d**, **e**, and **f**) areas

Representative specimens used for calculating the percentage of new bone area in the substitute at each examined site are shown in Figure 8. Average new bone formation within UDPHAp was significantly greater at the cortical bone side (43.3% ± 4.2%) compared to the medullary side (31.0% ± 2.4%) at 52 weeks (*p* = 0.0002). A similar trend was observed for new bone formation in the two regions at 104 weeks (37.0% ± 9.4% and 28.3% ± 7.2%, respectively; *p* = 0.0445). There was no difference in bone formation area at the cortical sides between 52 and 104 weeks.

We previously reported that bone ingrowth and capillary formation within UDPHAp bone substitute was observed as early as two weeks after implantation and was maintained for up to 12 weeks [6]. Karageorgiou and Kaplan [7] found that pore diameters greater than 50 µm are favorable for cell migration. Chang *et al.* [8] reported that the formation of bone and marrow in a hydroxyapatite bone substitute were greater in material with 300–500-µm pores than that in material with 50–100-µm pores. The 100~300-µm pore diameter and 75% porosity in UDPHAp also clearly supported cell migration and promoted bone regeneration and remodeling, suggesting that this material has the potential to be an effective bone substitute material for the repair of cortical bone defects [9]. Notably, the osteocyte canaliculi that form within the bone substitute may contribute to differences in the degree of bone formation between cortical and medullary regions by sensing the mechanical environment through the mechanosensory system that functions in bone. Our results suggest that mechanical stress from cortical bone or medullae and the interaction among osteogenetic, fibrous and hematopoietic cells may control osteogenesis inside the UDPHAp bone substitute [10,11].

In the present study, we also found that part of the implanted artificial bone was resorbed or replaced by autologous tissue 104 weeks after implantation into the rabbit tibial defect model. This finding is consistent with a study by Matsumine *et al.* [12], who showed that hydroxyapatite bone substitute inserted into cortical and cancellous defects created by the removal of a benign tumor was gradually resorbed. Hydroxyapatite is not generally considered to undergo resorption by osteoclasts, as the resorption of hydroxyapatite occurs by hydrolysis at a rate of a few microns per year. In the present UDPHAp samples, submicron granules of hydroxyapatite were observed at high magnification. The biological compatibility of hydroxyapatite crystals with bone and bone marrow has been demonstrated in animal experiments. For example, Rampel *et al.* [13] demonstrated that hydroxyapatite is directly resorbed by osteoclasts; however, this finding has not been confirmed in long-term studies with animal bone defect models. In addition, Akazawa *et al.* [14,15] reported that the resorption rate of hydroxyapatite bone substitute is controllable by biomimetic and chemical modifications, such as the use of specific dissolution-precipitation technique and supersonic treatment to rapidly increase the resorption rate compared to commercially available Hap ceramics. Recently, Guihard and Vicky [16,17] demonstrated that monocyte/macrophages can induce mesenchymal stem cells to undergo osteogenesis. However, the underlying mechanisms of these phenomena require further verification.

A few limitations of this study warrant mention. First, because the number of the specimens was relatively small and only two time points were examined, larger-scale studies are needed to confirm the present results. Second, the tibial bone defect was not of a critical size; therefore, the sham-operated animals would have been able to heal without the implantation of bone substitute. Despite these limitations, the present findings demonstrate that the unidirectional architecture of the UDPHAp pore structure promotes the formation and remodeling of bone at cortical sites of bone defects.

Using a rabbit tibial defect model, we showed that new bone tissue is formed within UDPHAp blocks implanted at bone defect sites and that the formed tissue undergoes continuous remodeling for up to two years. The reason for using a non-critical cortical defect was to determine differences in bone formation and remodeling between cortical and medullary lesions. Notably, the substitution of autologous bone was greatest in the regions of the bone substitute that were continuous to the cortical bone, and even after two years, the observed differences in bone formation in the cortical and medullary canals were maintained. Histological and immunological analyses showed that osteoblasts, osteocytes and capillaries were present in the newly-formed bone tissue within the implanted UDPHAp blocks. In addition, cathepsin K-positive cells were more common in the medullary luminal regions of the defect. Taken together, these findings suggest that continuous long-term bone remodeling occurs within UDPHAp implanted at bone defect sites by coupling osteogenesis and angiogenesis [7].

## 3. Experimental Section

### 3.1. Bone Defect Animal Model

**Figure 9:**
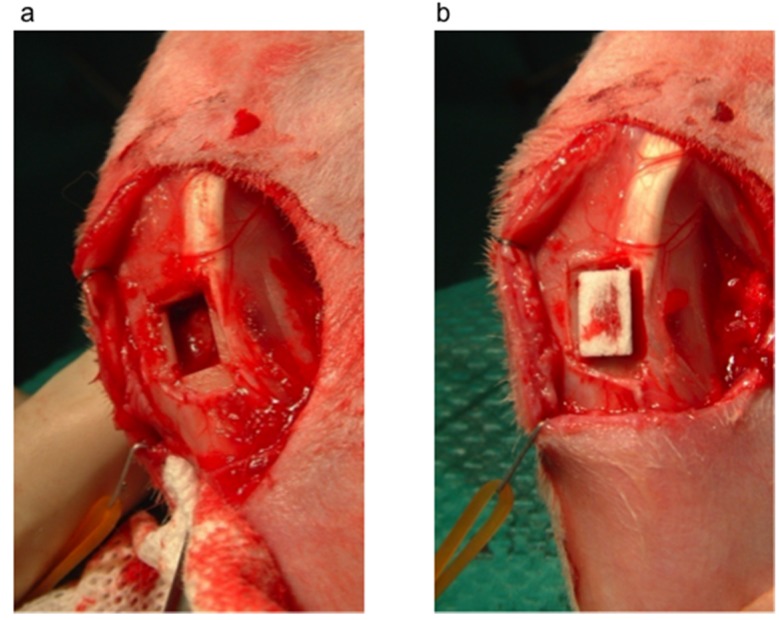
Creation of a cortical bone defect model and implantation of UDPHAp. (**a**) A rectangular-shaped cortical bone defect was created in the proximal tibia of a Japanese white rabbit after the removal of periosteum; (**b**) A trapezoidal-shaped UDPHAp block was implanted into the defect site.

**Figure 10:**
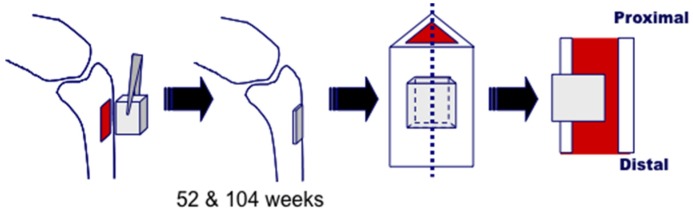
Schematic diagram of the implantation procedure and preparation of samples for histological analysis.

Under intravenous injection of pentobarbital (32 mg/kg, Somnopentil^TM^), a rectangular-shaped cortical bone defect (4.5 mm × 7 mm) was created in the proximal tibia of 8 Japanese white rabbits (20–23 weeks old) after removal of the periosteum (Figure 9a). A trapezoidal-shaped UDPHAp block (Figure 10) with 75% porosity was press-fitted into the tibial cavity (Figure 9b). The UDPHAp was made by a freeze-casting method and obtained by the Kuraray Co., Ltd. (Tokyo, Japan) [3]. The pore direction of the implanted UDPHAp block was parallel to the long axis of the tibia. After implantation, the surface of the UDPHAp block protruded approximately 1 mm from the surface of the cortical bone. At 52 and 104 weeks after implantation, 4 rabbits were euthanized by the intravenous injection of pentobarbital (100 mg/kg), and the implanted UDPHAp blocks and surrounding host bone with soft issues were harvested for histological analysis. A schematic diagram of the experimental outline is shown in Figure 10. The breeding of rabbits was performed in separated cages following the guidelines. Sham surgery to create the tibial defect without UDPHAp block implantation was performed for 5 rabbits (52-week (*n* = 2) and 104-week recovery (*n* = 3)). Three other rabbits with a cortical defect were left open for control (52-week (*n* = 2) and 104-week recovery (*n* = 1)). All animal experiments were performed under the approval of the ethical committee of the University of Tsukuba.

### 3.2. Histological Analysis

Decalcified slices of the collected UDPHAp samples were used for hematoxylin-eosin, osteocalcin, cathepsin K and Bodian staining. In addition, undecalcified ground sections of the UDPHAp samples were examined using Villanueva–Goldner staining to determine the area of bone within the substitute.

### 3.3. Bone Formation in Cortical and Medullary Bone Regions

**Figure 11:**
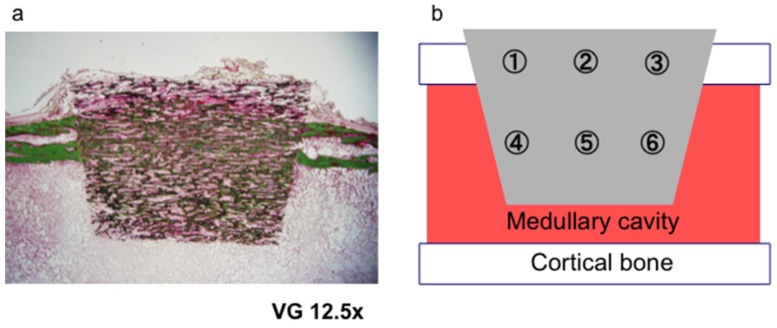
Sites used for the analysis of new bone formation in the cortical and medullary areas of the implanted UDPHAp block (**a**). The bone-occupied area inside UDPHAp was calculated at three fields (100× magnification) for both cortical bone regions (1–3) and the intramedullary (4–6) in each one section (**b**).

Bone-occupied area inside UDPHAp was calculated at three fields (100× magnification) for both the intramedullary (1–3) and cortical bone regions (4–6) in each section (Figure 11). New bone area within the UDPHAp was calculated with image analysis software (Mac Scope). Three fields per sample in the cortical and medullary regions (6 sites for 52-week samples and 9 sites for 104-week samples) were evaluated using an optical microscope under 100× magnification, and the localization of new bone was compared between the different sites in sections of the transplanted UDPHAp blocks.

### 3.4. Statistical Analysis

Differences of the bone-formation ratio between cortical and medullae were evaluated using the Welch test. All statistical analyses were performed using the Statcell software (Version 2; OMS Ltd., Saitama, Japan). A *p*-value < 0.05 was considered statistically significant.

## 4. Conclusions

Bone formation and remodeling was observed within UDPHAp blocks in a cortical defect site of rabbit tibias for up to two years after implantation. Notably, the percentage of newly-formed bone area was larger in cortical lesions than that in medullary lesions, indicating that tissue surrounding the defect site has mechanical and/or chemical effects on the remodeling process. These findings suggest that UDPHAp is potentially suitable for the repair of non-critical-sized cortical bone defects in the clinical setting.
